# Go West, Early Man: Modeling the Origin and Spread of Early Agriculture

**DOI:** 10.1371/journal.pbio.0030436

**Published:** 2005-11-29

After the last major ice age some 10,000 years ago, things began to look up for early humans. Forbidding climes yielded to more hospitable weather patterns, and people began to settle down and domesticate plants and animals. Archeologist Gordon Childe, who in 1942 called the transition from hunting and gathering to agriculture the Neolithic Revolution, proposed that unchecked population growth triggered economic and social problems among Near Eastern populations and forced farmers and shepherds to search for new lands. In this demic diffusion model, dispersing populations introduced Europeans to the Neolithic lifestyle. Alternately, Europeans may have learned to farm by imitating Neolithic practitioners they encountered through trade or other interactions (the cultural diffusion model).

Childe's ideas of westward migration found support in a 1965 study that mapped the spatiotemporal pattern of a small sample of radiocarbon dates (determined from animal bones and other carbon remains) from Neolithic sites. A landmark study by Albert Ammerman and Luigi Cavalli-Sforza in 1971 used more data—radiocarbon dates from 53 early Neolithic sites—and used a population biology model to investigate Neolithic spread. Their “wave of advance” model proposed that population growth at the agricultural fringes coupled with local migrations would produce steady population expansions in all directions. They calculated an average rate of spread of about one kilometer per year.

But the controversy between the cultural and demic diffusion models still remains today. Now, over 30 years later, Ron Pinhasi and Joaquim Fort revisited the question along with Ammerman, using a substantially larger dataset with new locations—radiocarbon-dated bones and charcoal from 735 Neolithic sites in Europe, the Near East, and Asia—and reaffirm the wave-of-advance model. The authors combined mathematical and geospatial techniques to estimate the timing and likely center of agricultural origins, as well as the rate of spread. Their results support a model of demic diffusion and, for the first time, pinpoint the geographic origin of agriculture within the Fertile Crescent.

Pinhasi et al. calculated the correlation between the straight distance versus age of the 735 radiocarbon dates and the likely spread from 25 hypothetical centers of origin (based on location only) and ten probable centers (sites that included the oldest remains, as well as a center proposed in the 1971 study). The most southern point, Abu Madi in Egypt, had the highest correlation, though eight of the other probable centers had similar scores. However, charting the shortest paths (which take into account the barrier effect of the Mediterranean Sea), pointed to an origin in the north. Focusing on the centers that seemed most likely, Pinhasi et al. used both approaches (one based on straight paths, one based on shortest paths) to estimate the speed of agricultural spread, and came up with nearly the same figure: 0.7–1.1 kilometers per year versus 0.8–1.3 kilometers per year. An error range for this speed was estimated (which had not been done before), so the authors could also compare this observed rate with that predicted by a model.

While no cultural diffusion model is known so far that can explain the observed rate (calculated from the archeological evidence), a kilometer or so a year is consistent with a time-delayed demic diffusion model. (This model, which was proposed by Fort and co-workers in 1999, also agrees with data from other human and nonhuman population expansions, as well as with the observed speeds of virus infections.) While many genetic studies also support demic diffusion, they do not agree on the extent to which Near Eastern farmers contributed to the European gene pool. Assuming a linear advance, agricultural expansion began some 9,000–11,500 years ago, falling in line with a gradual wave of advance. Rather than “racing across the map of Europe,” the authors argue, the Neolithic transition took over 3,000 years, or 100 generations, reflecting the time children stay with their parents before moving on to greener pastures. This is precisely the time-delay effect that classical diffusion models are unable to capture, but that is accounted for in the model by Fort and co-workers. Finally, the authors incorporated radiocarbon data from 30 sites in Arabia to find the most likely birthplace of agriculture. Their shortest-path analysis points to northern Levant and northern Mesopotamia (whereas the straight-path, or classical, approach pointed to a southern origin).

The authors' approach did not address whether migrants traveled by land or by sea or whether farmers displaced foragers. But the pattern and processes of dispersal were likely complex, Pinhasi et al. conclude, with multiple paths and mechanisms fueling the western expansion of the Neolithic lifestyle. And with a newly bolstered wave-of-advance model and the approach outlined here, geneticists, anthropologists, and other researchers investigating the origin and spread of human populations have a more detailed roadmap to follow.

*Note for international readers: During the time of Westward expansion in the 19th century, American essayist Horace Greeley famously advocated Manifest Destiny by exhorting, “Go West, young man!”* —*Liza Gross*

